# Supplementary material for: Structural basis for high selectivity of a rice silicon channel Lsi1
Source: Nat Commun. 2021 Oct 29;12:6236. doi: 10.1038/s41467-021-26535-x (PMC8556265; doi:10.1038/s41467-021-26535-x)
Supplement: Supplementary file 1 — Supplementary Information [file 41467_2021_26535_MOESM1_ESM.pdf]

Supplementary Information:

Structural basis for high selectivity of a rice silicon channel Lsi1

Yasunori Saitoh, Namiki Mitani-Ueno, Keisuke Saito, Kengo Matsuki, Sheng Huang, Lingli Yang, Naoki Yamaji, Hiroshi Ishikita, Jian-Ren Shen, Jian Feng Ma*, and Michihiro Suga*

Contents:

Supplementary Figure 1. Amino acid sequence alignment of silicic acid channels and non-silicic acid channels.

Supplementary Figure 2. The construct $Lsi1_{cryst}$.

Supplementary Figure 3. FSEC profiles and substrate permeation ability of the Lsi1 constructs.

Supplementary Figure 4. Comparison of the structures of Lsi1 and other AQPs.

Supplementary Figure 5. Interactions of a water molecule between TM4 and TM5, and extensive interactions in loop C.

Supplementary Figure 6. Desolvation of $Si(OH)_4$ molecules on the vestibules in the 450 ns equilibrium MD simulation.

Supplementary Figure 7. Western blotting analysis of the wildtype and mutants expressed with *Xenopus oocytes* and Sf9 cells for Fig. 5.

Supplementary Figure 8. Effect of the selectivity filter mutations of aquaglyceroporin on the transport activity for glycerol, water, Ge, and As.

Supplementary Figure 9. QM/MM calclulation of Lsi1 with the modeled silicic acid.

Supplementary Figure 10. Water molecules and silicic acid found in the channel during the MD simulation.

Supplementary Figure 11. MD trajectory of the permeated silicic acid in the 450 ns simulation.

Supplementary Figure 12. Channel diameters and its profile of the $Lsi1_{cryst}$ structure.

Supplementary Figure 13. Comparison of the structures of Lsi1 and SoPIP2;1.

Supplementary Figure 14. Comparison of the structures of Lsi1 and AtTIP2;1.

Supplementary Figure 15. Comparison of the selectivity filters of Lsi1 and AtTIP2;1.

Supplementary Figure 16. Comparison of the structures of Lsi1 and hAQP10.

Supplementary Table 1. Substrate permeability of AQP family proteins.

Supplementary Table 2. Average number and average exchange time of water molecules on the site.

Supplementary Table 3. Primer list

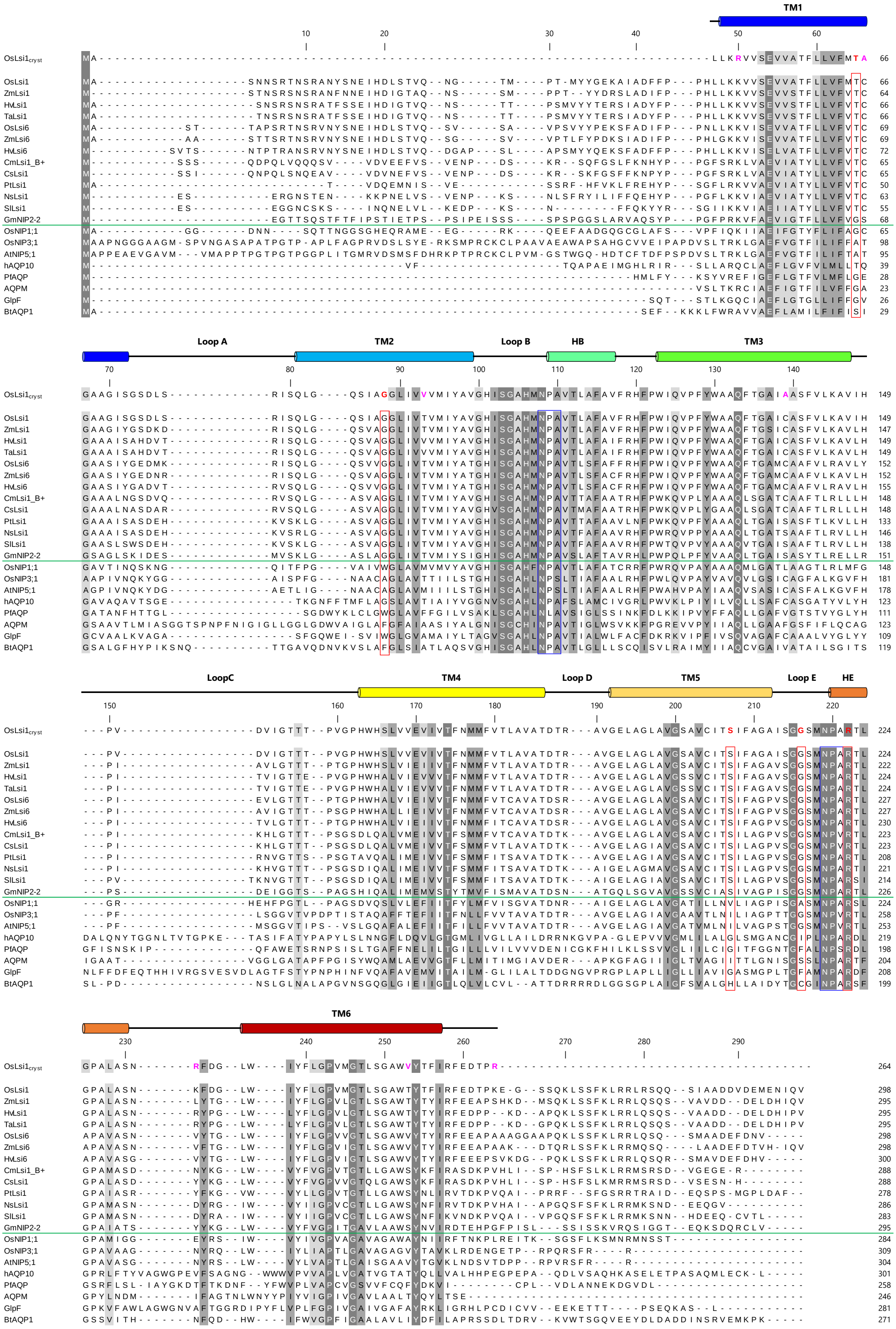
TM1
Loop A
TM2
Loop B
HB
TM3
LoopC
TM4
Loop D
TM5
Loop E
HE
TM6
OsLsi1cryst
OsLsi1
ZmLsi1
HvLsi1
TaLsi1
OsLsi6
ZmLsi6
HvLsi6
CmLsi1_B+
CsLsi1
PtLsi1
NsLsi1
SlLsi1
GmNIP2-2
OsNIP1;1
OsNIP3;1
AtNIP5;1
hAQP10
PfAQP
AQPM
GlpF
BtAQP1

Supplementary Figure 1 | Amino acid sequence alignment of silicic acid channels and non-silicic acid channels. Amino acid sequence alignment of the OsLsi1$_{cryst}$ construct, OsLsi1 (XP_015626173.1) and other homologues ZmLsi1 (NP_001105637.1), HvLsi1 (BAH24163.1), TaLsi1 (ADM47602.1), OsLsi6 (XP_015644134.1), ZmLsi6 (NP_001105517.1), HvLsi6 (BAH84977.1), CmLsi1_B+ (BAK09175.1), CsLsi1 (NP_001267699.1), PtLsi1 (XP_002324057.1), NsLsi1 (XP_009801656.1), SlLsi1 (NP_001274283.1), GmNIP2-2 (NP_001240190.1), OsNIP1;1 (XP_015623151.1), OsNIP3;1 (XP_015614995.1), AtNIP5;1 (NP_192776.1), hAQP10 (NP_536354.2), PfAQP (CAC88373.1), AQPM (WP_013295370.1), GlpF (NP_418362.1), and BtAQP1 (NP_777127.1). Highly conserved residues are highlighted in a grey background, with the darkness indicating the degree of conservation. Silicic acid channels are listed above the green line. The residues comprising the selectivity filter, and the NPA motifs are surrounded by red and blue lines, respectively. In the OsLsi1$_{cryst}$ sequence, the residues comprising the selectivity filter and point mutations are shown in red and magenta, respectively.

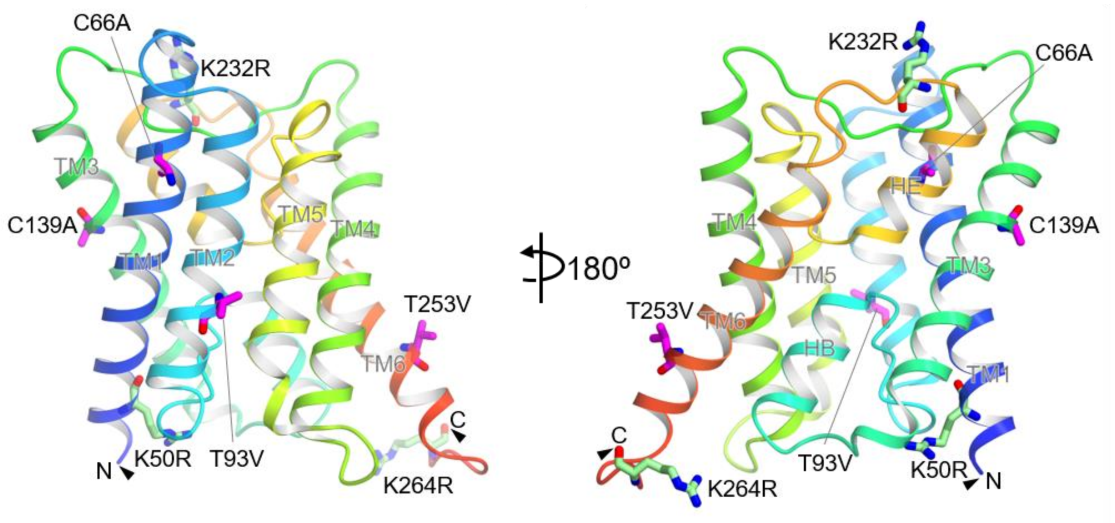

Supplementary Figure 2 | The construct Lsi1$_{\mathbf{cryst}}$. Side views of the Lsi1$_{cryst}$, rainbow-colored with the N terminus in blue. Arrowheads indicate the truncation sites of N and C terminus. Point mutations which enhanced the thermo-stability of Lsi1 (C66A, T93V, C139A and T253V) are colored in magenta, and those likely reduce the surface entropy of Lsi1 (K50R, K232R and K264R) are colored in light green.

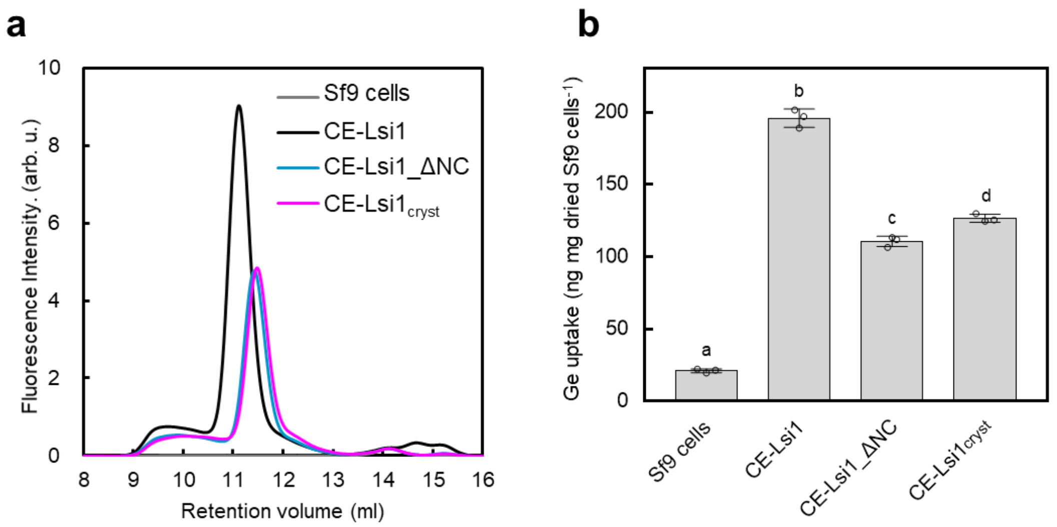

Supplementary Figure 3 | FSEC profiles and substrate permeation ability of the Lsi1 constructs. a, FSEC profiles of solubilized Sf9 cells expressing the EGFP-tagged full length Lsi1 (CE-Lsi1) and Lsi1 mutants (CE-Lsi1_ΔNC, and CE-Lsi1$_{cryst}$). **b,** Germanic acid conductivity of Sf9 cells expressing CE-Lsi1, CE-Lsi1_ΔNC, and CE-Lsi1$_{cryst}$. Data are means ±s.d., n = 3 independent experiments. Different letters above the columns indicate statistically significant differences at $P <$ 0.01 by Tukey's test, and test was two-sided. In **a**, **b**, Sf9 cells without infection were analyzed as a control.

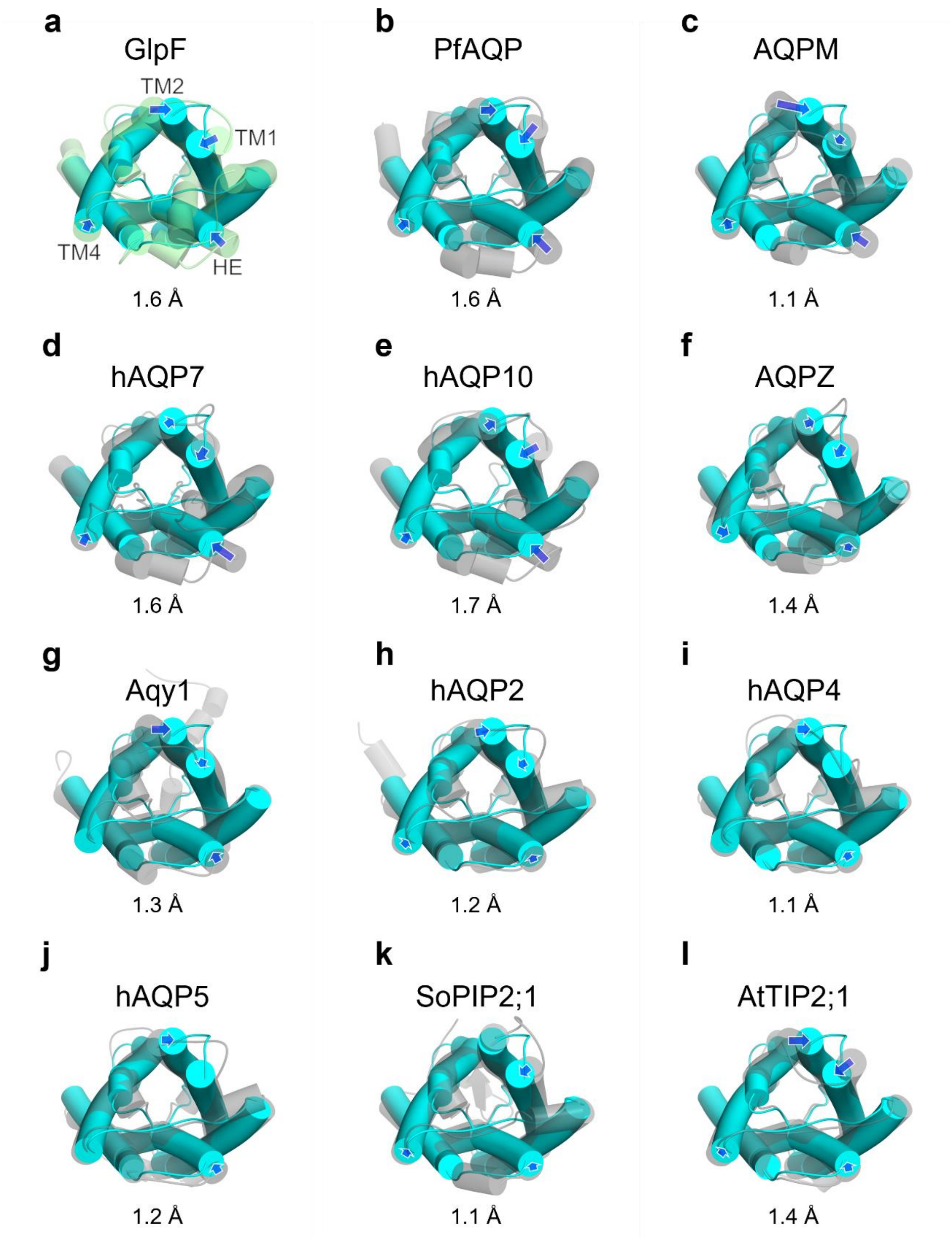

Supplementary Figure 4 | Comparison of the structures of Lsi1 and other AQPs. Superposition of the structure of Lsi1$_{cryst}$ (cyan) with other AQPs (green or gray), GlpF (**a**), PfAQP (**b**), AQPM (**c**), hAQP7 (**d**), hAQP10 (**e**), AQPZ (**f**), Aqy1 (**g**), hAQP2 (**h**), hAQP4 (**i**), hAQP5 (**j**), SoPIP2;1 (**k**), and AtTIP2;1 (**l**). The RMSD values are shown in Å. View directions are same with Fig. 2a.

a

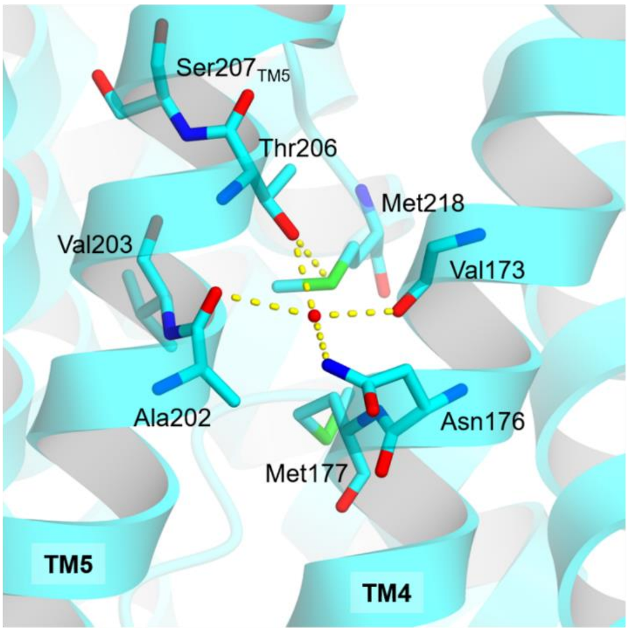

b

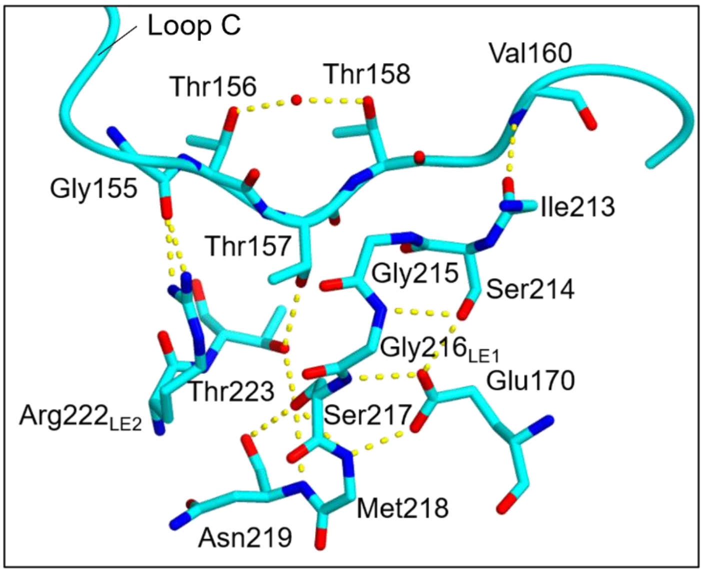

Supplementary Figure 5 | Interactions of a water molecule between TM4 and TM5 (a), and extensive interactions in loop C (b). Amino acid residues interacting with each other or with water molecules (red spheres) are represented as stick model. Hydrogen bonding interactions and polar interactions are shown in yellow dashes.

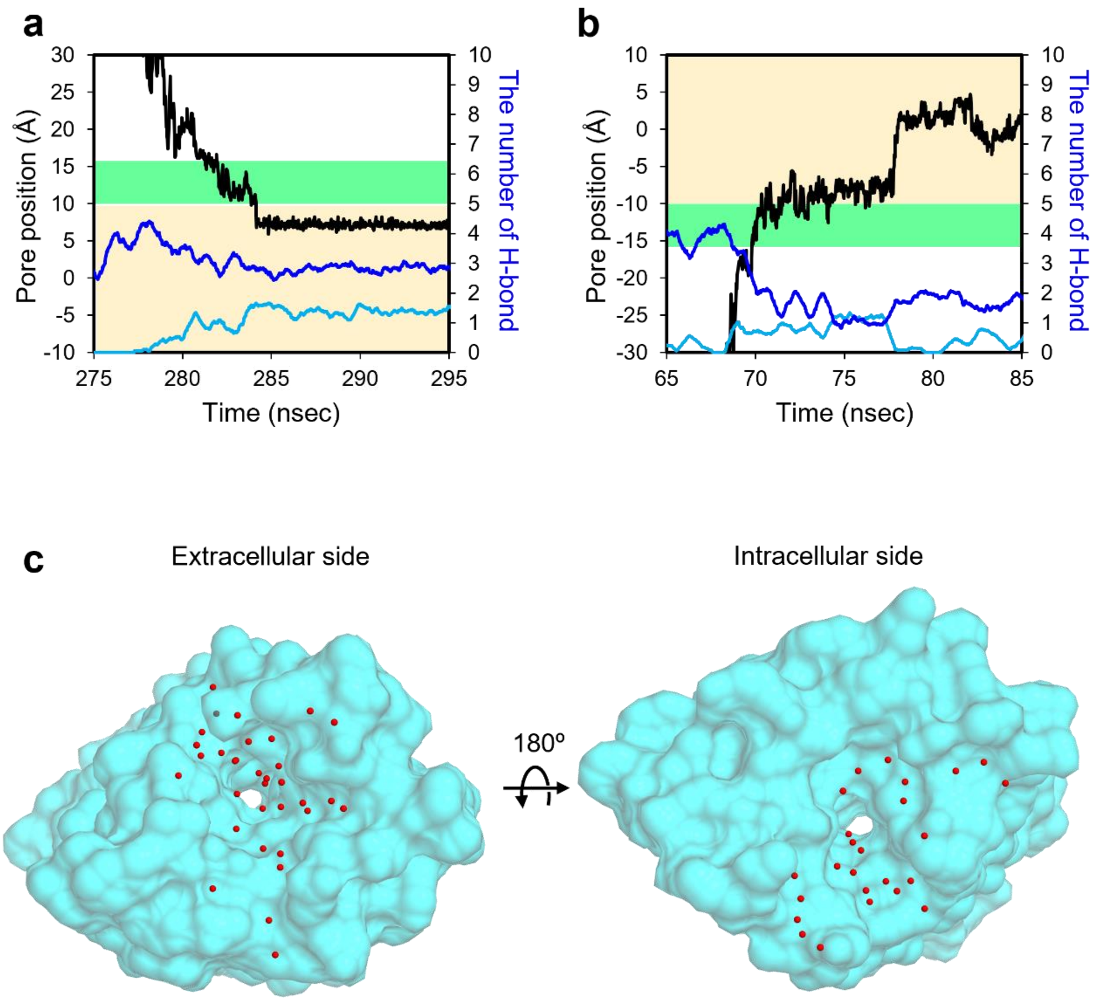

Supplementary Figure 6 | Desolvation of $Si(OH)_4$ molecules on the vestibules when $Si(OH)_4$ enters from (a) the extracellular side and (b) the intracellular side in the 450 ns equilibrium MD simulation. Black line shows the trajectory (the z coordinate) of $Si(OH)_4$ (measured in the left axis). Blue and cyan lines show numbers of H-bonded water molecules and H-bonded amino acids of the protein, respectively (measured in the right axis). The vestibules and channel region are colored in light green and khaki, respectively. (**c**) Water molecules at the extra/intracellular regions found in the crystal structure.

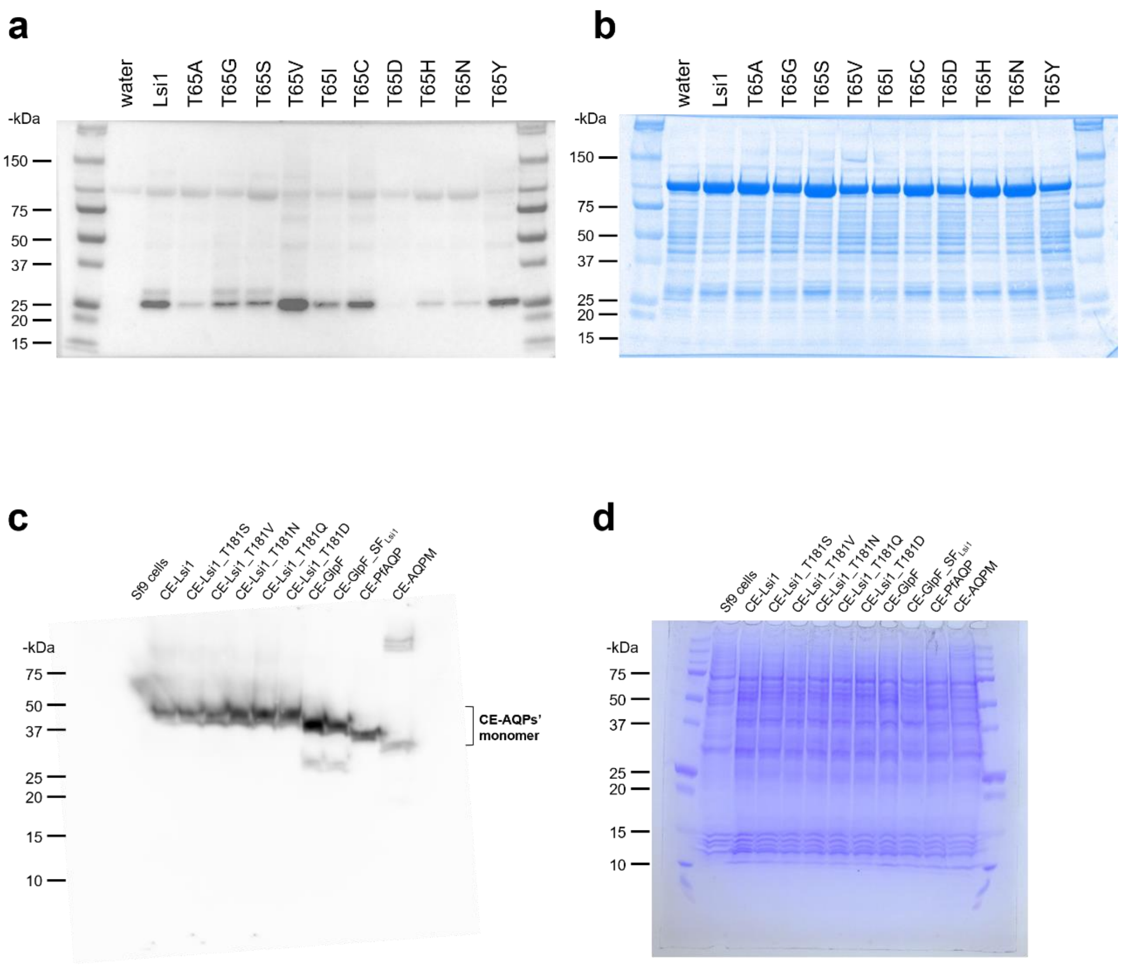

Supplementary Figure 7 | Western blotting analysis of the wildtype and mutants expressed with *Xenopus oocytes* (a, b) and Sf9 cells (c, d) for Fig. 5. Western blotting signals were detected using mouse monoclonal ANTI-FLAG® M2-HRP antibody (**a**) or anti-green fluorescence protein polyclonal antibody-HRP-DirecT (**c**). CBB-staining of SDS-PAGE (**b, d**). These experiments were done once (**a** and **b**) or repeated twice with similar results (**c** and **d**).

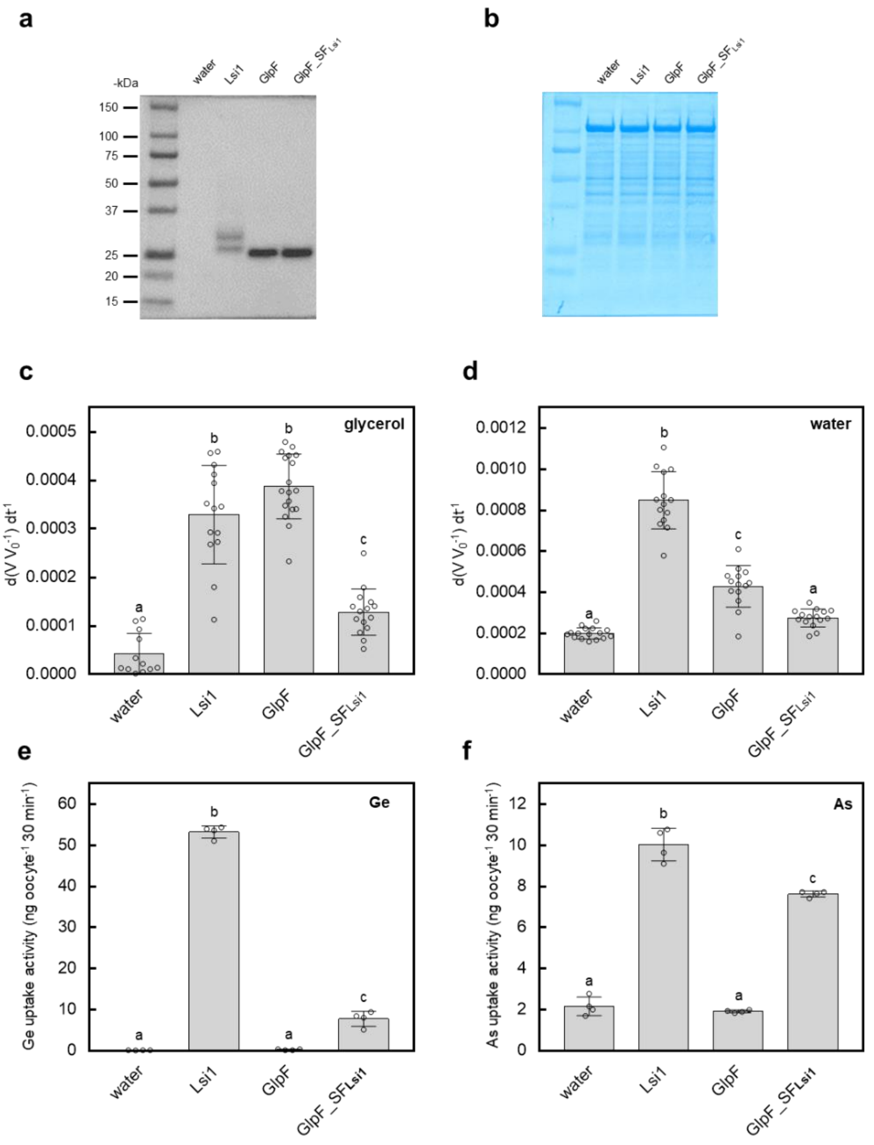

Supplementary Figure 8 | Effect of the selectivity filter mutations of aquaglyceroporin on the transport activity for glycerol, water, Ge, and As.

a and **b**, Western blotting and CBB staining of SDS-PAGE with *Xenopus* oocytes used in the experiments (**c**-**f**). **c**-**f**, Transport activity of glycerol (**c**), water (**d**), Ge (**e**), and As (**f**) in *Xenopus* oocytes. The substrate uptake experiments were carried out in a concentration gradient for **c** and **d** (170 mM glycerol and 5-fold diluted MBS). In (**c**-**f**), different letters above the columns indicate statistically significant differences at $P < 0.05$ by Tukey-Kramer's test, and test was two-sided. Values are means ± s.d., **c**: n = 12 (water), n = 14 (Lsi1), n = 15 (GlpF_SF$_{Lsi1}$), n = 18 (GlpF); **d**: n = 14 (Lsi1, GlpF), n = 15 (GlpF_SF$_{Lsi1}$), n = 16 (water); **e** and **f**: n = 4; independent experiments.

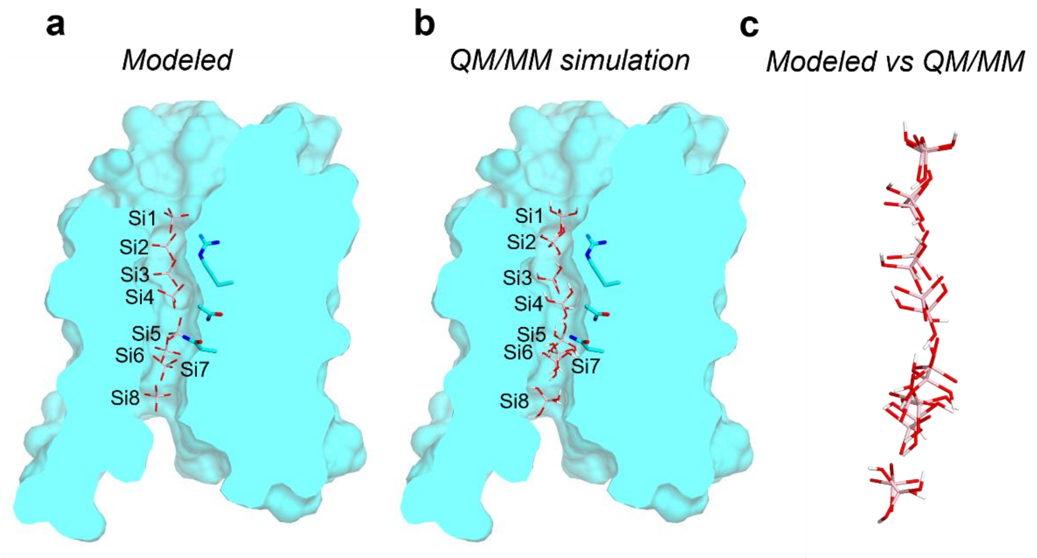

d

	Si-Si distance (Å)	The no. of hydrogen bonds		
		Original	QM/MM	Δ
**Si1**	0.2	5 (2.7)	5 (2.9)	0
**Si2**	0.5	9 (3.0)	8 (2.8)	1
**Si3**	0.8	5 (2.7)	6 (2.8)	-1
**Si4**	0.9	4 (2.7)	3 (2.9)	1
**Si5**	0.8	4 (3.0)	4 (3.0)	0
**Si6**	0.7	3 (2.8)	4 (3.0)	-1
**Si7**	0.7	2 (2.9)	2 (3.0)	0
**Si8**	0.8	2 (2.8)	4 (3.1)	-2

Supplementary Figure 9 | QM/MM calculation of Lsi1 with the modeled silicic acid.

The modeled $Si(OH)_4$ molecules (**a**), and the $Si(OH)_4$ molecules obtained from the QM/MM calculation (**b**), and their superposed structure (**c**). **d**, A comparison with respect to the displacement of the Si atoms in Å, the number of hydrogen bonds with Lsi1, and the average hydrogen bond distances (values in brackets in Å).

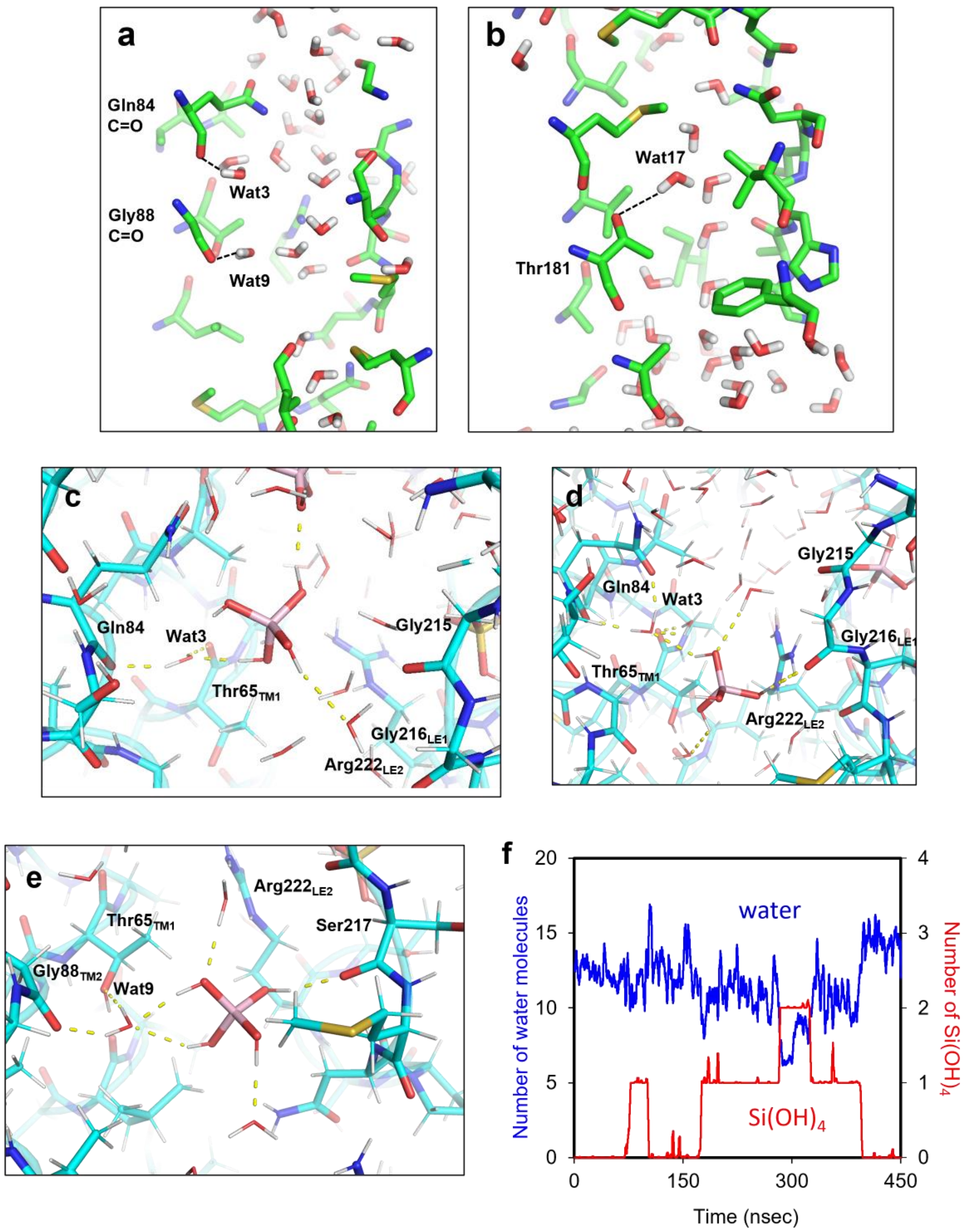

Supplementary Figure 10 | Water molecules and silicic acid found in the channel during the MD simulation. MD snapshots of the water molecule Wat3 and Wat9 (**a**), and Wat17 (**b**). MD snapshots showing the interactions between silicic acid and Wat3 (**c**, **d**) and between silicic acid and Wat9 (**e**). **d** and **e** are the snapshots of the permeated silicic acid. **f**, Number of the water molecules (blue) and the $Si(OH)_4$ molecules (red) found in the channel during the simulation. The number is obtained from the moving average over the 2-ns window in the region of -7.6 Å < pore position < 10 Å. The definition of the pore position is the same as Fig. 2d.

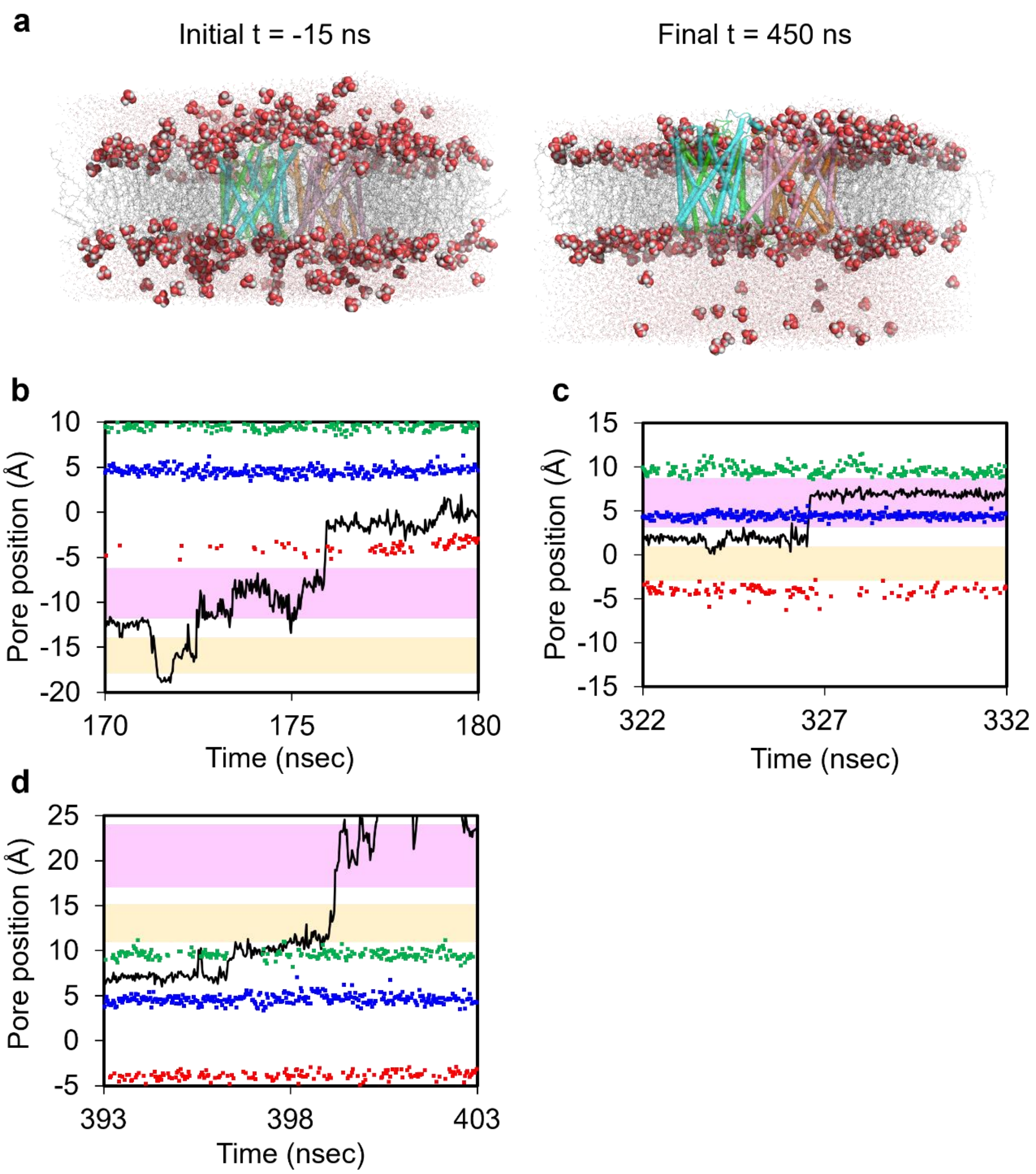

Supplementary Figure 11 | Distributions of $Si(OH)_4$ molecules and trajectory of the permeated silicic acid in the 450 ns simulation. (**a**) The positions of $Si(OH)_4$ molecules at the beginning (left) and end (right) of the simulation. The trajectory of the silicic acid permeation during the 170 - 180 ns (**b**), 322-332 ns (**c**), and 393-403 ns (**d**). Positions of silicic acid (black), Wat3 (green), Wat9 (blue), Wat17 (red) in the channel are plotted. The regions for SF and NPA motifs are colored in plum and khaki, respectively. The trajectory during 100-450 ns is provided as Fig. 7**f**.

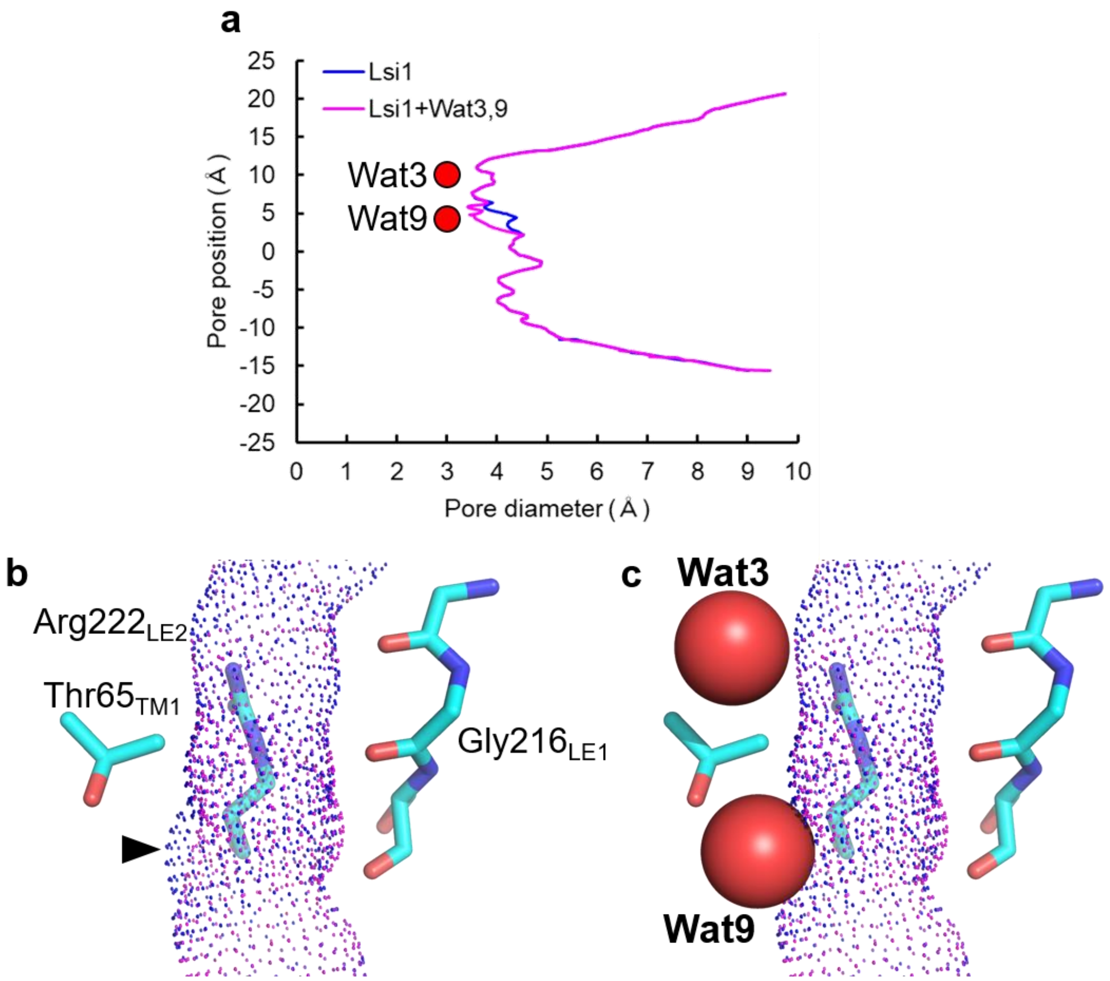

Supplementary Figure 12 | Channel diameters and its profile of the Lsi1$_{\mathbf{cryst}}$ structure. Channel diameters are calculated with (pink) and without (blue) Wat3 and Wat9 in the structure (**a**). The selectivity filter's close-up views are shown without (**b**) and with (**c**) Wat3 and Wat9. The structural bottleneck made by Wat9 is indicated by an arrowhead.

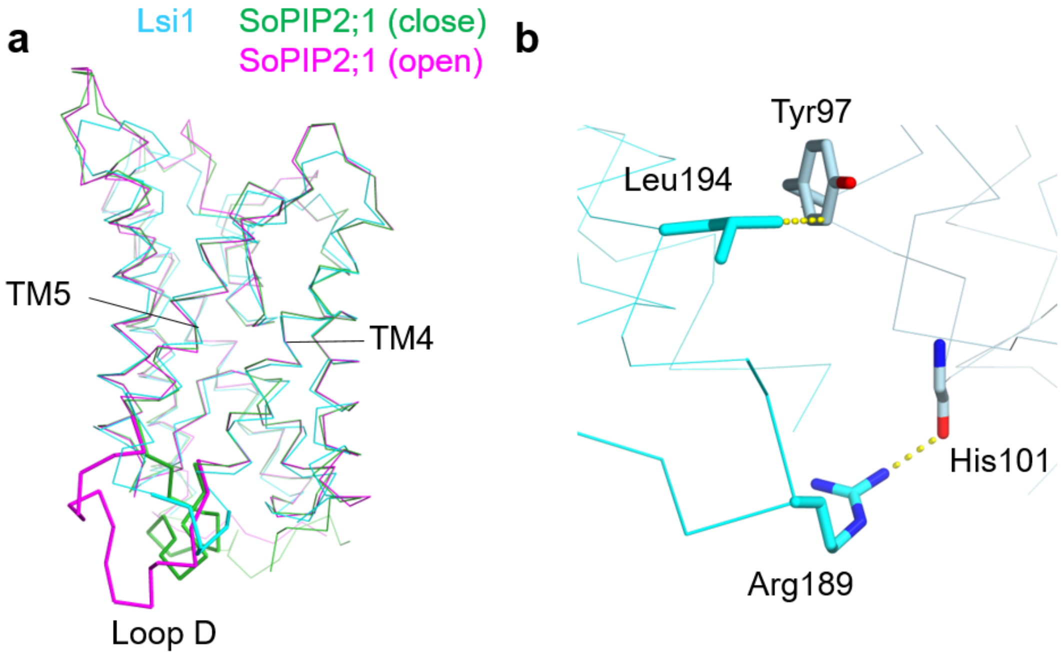

Supplementary Figure 13 | Comparison of the structures of Lsi1 and SoPIP2;1.

Superposition of the structure of Lsi1$_{cryst}$ (cyan) with SoPIP2;1 (PDB 1Z98 close conformation, lime green; PDB 2B5F, open conformation, magenta) (**a**). Loop D is highlighted in bold sticks. Interaction between the loop D and adjacent monomer's TM2 in the Lsi1 structure (**b**). In (**b**), chains A and B are colored in cyan and light blue, respectively.

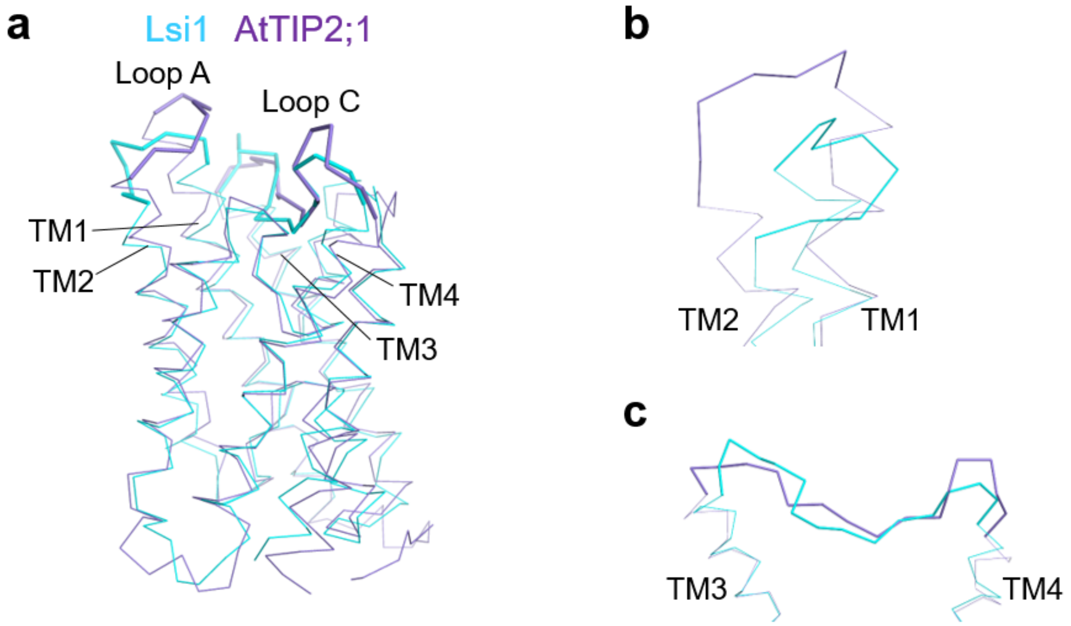

Supplementary Figure 14 | Comparison of the structures of Lsi1 and AtTIP2;1.

Superposition of the structure of Lsi1$_{cryst}$ (cyan) with AtTIP2;1 (PDB 5I32, light purple) (**a**). Loop A and C are highlighted in bold sticks. Close-up view of the loop A (**b**) and loop C (**c**).

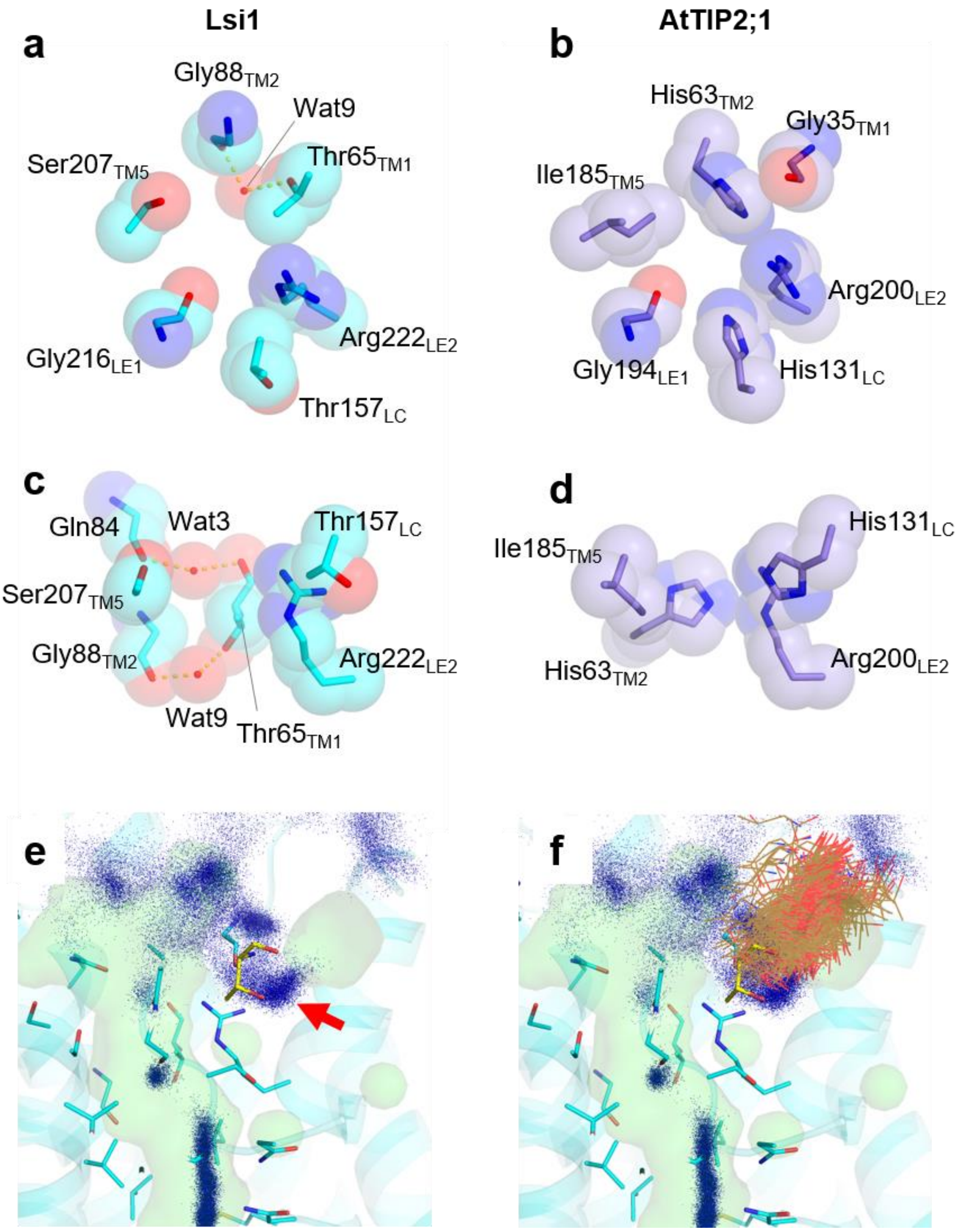

Supplementary Figure 15 | Comparison of the selectivity filters of Lsi1 and AtTIP2;1.

The selectivity filter of Lsi1$_{\mathrm{cryst}}$ (**a**, **c**, cyan), and AtTIP2;1 (**b**, **d**, light purple, PDB 5I32), are shown together with the additional residue from loop C. Top views (**a**, **b**) and side views (**c**, **d**) of the selectivity filter. In (**a**, **b**), the view direction is the same as Fig. 3. The Si atom positions of silicic acid (blue dots) during the 0-300 ns MD simulation (**e**, **f**). The Si atoms at Thr157 is indicated by a red arrow in (**e**), and Thr157 in the crystal structure is shown in a yellow stick in (**e**, **f**), and all Thr157 conformations during the MD simulation is overlaid in (**f**).

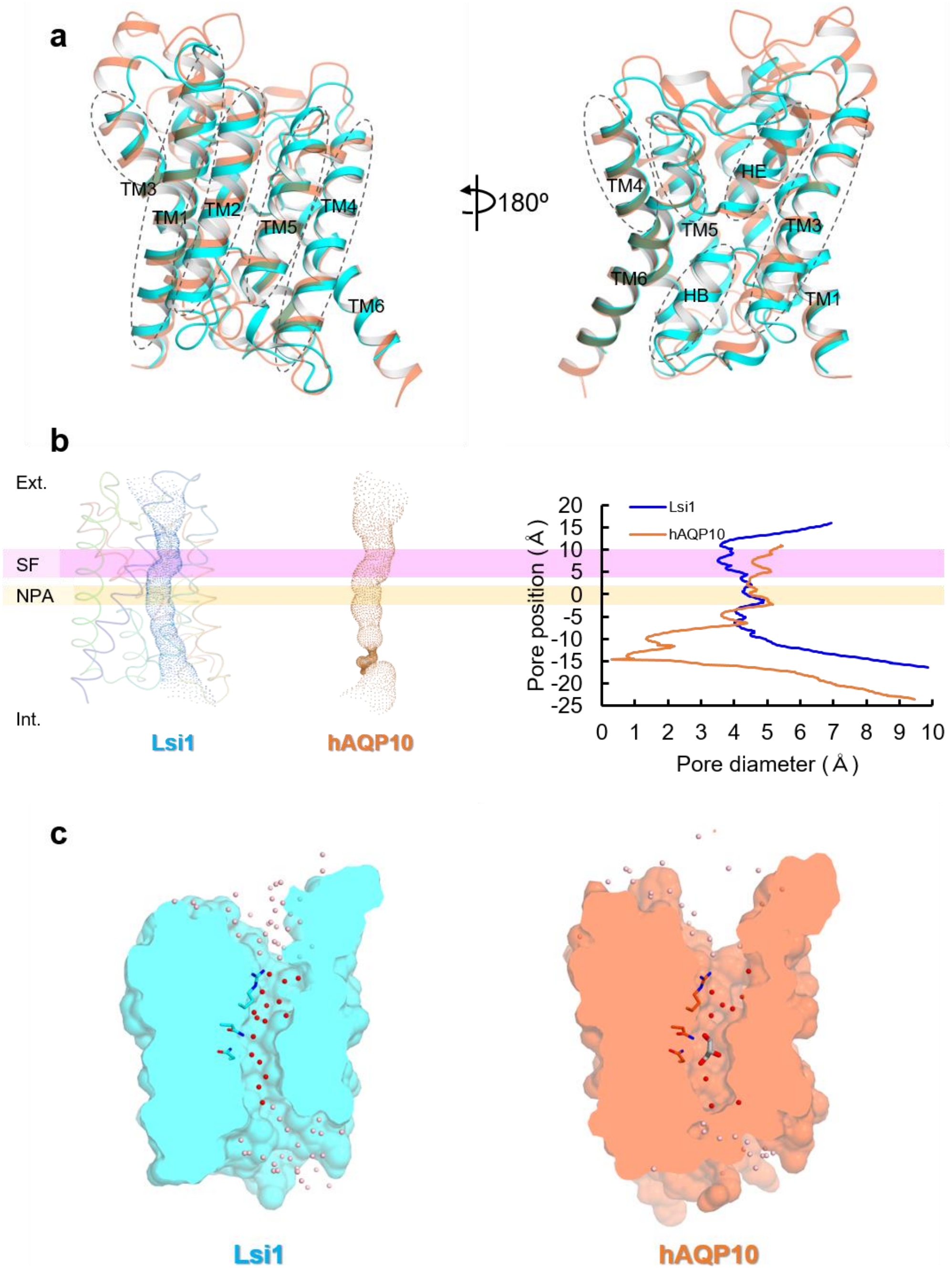

Supplementary Figure 16 | Comparison of the structures of Lsi1 and hAQP10.

a, Superposition of the structure of $Lsi1_{cryst}$ (cyan) with hAQP10 (PDB 6F7H, orange). Dotted circles indicate the areas where TM helices tilt in different orientations. **b**, Channel profile (left) and diameters (right) along the pore for $Lsi1_{cryst}$ and hAQP10. **c**, Cross-section of the channel for $Lsi1_{cryst}$ (left, cyan) and hAQP10 (right, orange).

Supplementary Table 1 | Substrate permeability of AQP family proteins

	Silicic acid	Arsenite	Boric acid	water	glycerol	Ammonia	Reference
Lsi1	+	+	+	+	+/-	?	This study, [1-3]
Mammalian AQP1	?	?	?	+	-	-	[4-5]
Aqy1	?	?	?	+	?	?	[6]
GlpF	-	+	?	+	+	?	This study, [4]
PfAQP	-	+	?	+	+	?	This study, [7]
AQPM	-	-	?	+	+	?	This study, [4]
hAQP10	+	?	?	+	+	?	[8-9]
SoPIP2;1	?	?	?	+	?	?	[4]
AtTIP2;1	?	?	?	+	?	+	[5]

Plus indicates permeable, and minus indicates impermeable. Plus/minus for Lsi1 means Lsi1 transports glycerol at a high concentration of 170 mM but does not transport at a low or physiological concentration of 2 mM. Question marks indicate no study has reported for the permeability.

Supplementary Table 2 | Average number and average exchange time of water molecules on the site.

	**Wat3[1]**	**Wat9[2]**	**Wat17[3]**
$\boldsymbol{n}_{\mathbf{av}}$	0.597	0.943	0.437
**τ (ns)[4]**	0.30	1.50	0.31

[1] Hydrogen bonded with C=O of Gln84. We defined the hydrogen bond when the O_{Wat}-O distance < 3.2 Å.

[2] Hydrogen bonded with C=O of Gly88.

[3] Hydrogen bonded with O of the sidechain of Thr181.

[4] Calculated by $\tau = T\, n_{ave} / N_{wat}$, where T is the simulation time 450 ns, and N_{wat} is the number of unique water molecules on the site during the simulation.

Supplementary Table 3 | Primer list.

Name	Sequence
**CE-Lsi1 delN_3-40A_f**	CATGGCCGACTTCTTCCCTCCTCAC
**CE-Lsi1 delN_3-40A_r**	AAGAAGTCGGCCATGGTGGCGGATCC
**CE-Lsi1 delC265_f**	CCCCAAGGAAAACCTGTATTTTCAG
**CE-Lsi1 delC265_r**	AGGTTTTCCTTGGGGGTGTCCTCGAA
**CE-Lsi1d3-40_d41-46_f**	CATGGCCCTCCTCAAGAGGGTCGTG
**CE-Lsi1d3-40_d41-46_r**	TGAGGAGGGCCATGGTGGCGGATCC
**CE-Lsi1_K50R_f**	CTCACCTCCTCAAGAGGGTCGTGTCGGAGGTG
**CE-Lsi1_K50R_r**	CACCTCCGACACGACCCTCTTGAGGAGGTGAG
**CE-Lsi1_C66A_f**	ATGACGGCTGGGGCGGCAGGGATCAGC
**CE-Lsi1_C66A_r**	CGCCCCAGCCGTCATGAACACCAGCAG
**CE-Lsi1_T93V_f**	GGTGGCCTCATCGTGGTGGTGATGATCTACGCC
**CE-Lsi1_T93V_r**	GGCGTAGATCATCACCACCACGATGAGGCCACC
**CE-Lsi1_C139A_f**	GCGATAGCCGCGTCGTTCGTGCTCAAG
**CE-Lsi1_C139A_r**	CGACGCGGCTATCGCTCCGGTGAACTG
**CE-Lsi1_K232R_f**	CGCTGGCGAGCAACAGGTTCGACGGCCTGT
**CE-Lsi1_K232R_r**	ACAGGCCGTCGAACCTGTTGCTCGCCAGCG
**CE-Lsi1_T253V_f**	CTCTCGGGAGCATGGGTCTACACCTTCATCCGC
**CE-Lsi1_T253V_r**	GCGGATGAAGGTGTAGACCCATGCTCCCGAGAG
**CE-Lsi1_K264R_f**	CGAGGACACCCCCAGGGAAAACCTGTATTTTCAG
**CE-Lsi1_K264R_r**	CTGAAAATACAGGTTTTCCCTGGGGGTGTCCTCG
