# Supplementary material for: Structural basis for high selectivity of a rice silicon channel Lsi1
Source: Nat Commun. 2021 Oct 29;12:6236. doi: 10.1038/s41467-021-26535-x (PMC8556265; doi:10.1038/s41467-021-26535-x)
Supplement: Supplementary file 3 — Description of Additional Supplementary Files [file 41467_2021_26535_MOESM3_ESM.pdf]

Description of Additional Supplementary Files

File name: Supplementary Data 1

Description: The coordinates of Si molecules obtained after the QM/MM calculation. Eight Si molecules are provided as supplementary dataset in the PDB format.
