# Supplementary material for: Structural basis for high selectivity of a rice silicon channel Lsi1
Source: Nat Commun. 2021 Oct 29;12:6236. doi: 10.1038/s41467-021-26535-x (PMC8556265; doi:10.1038/s41467-021-26535-x)
Supplement: Supplementary file 5 — Reporting Summary [file 41467_2021_26535_MOESM5_ESM.pdf]

nature research

Corresponding author(s): Jian Feng Ma
Michihiro Suga

Last updated by author(s): 9/29/2021

Reporting Summary

Nature Research wishes to improve the reproducibility of the work that we publish. This form provides structure for consistency and transparency in reporting. For further information on Nature Research policies, see our Editorial Policies and the Editorial Policy Checklist.

Statistics

For all statistical analyses, confirm that the following items are present in the figure legend, table legend, main text, or Methods section.

n/a	Confirmed	
☐	☒	The exact sample size (n) for each experimental group/condition, given as a discrete number and unit of measurement
☐	☒	A statement on whether measurements were taken from distinct samples or whether the same sample was measured repeatedly
☐	☒	The statistical test(s) used AND whether they are one- or two-sided<br>*Only common tests should be described solely by name; describe more complex techniques in the Methods section.*
☒	☐	A description of all covariates tested
☒	☐	A description of any assumptions or corrections, such as tests of normality and adjustment for multiple comparisons
☐	☒	A full description of the statistical parameters including central tendency (e.g. means) or other basic estimates (e.g. regression coefficient) AND variation (e.g. standard deviation) or associated estimates of uncertainty (e.g. confidence intervals)
☐	☒	For null hypothesis testing, the test statistic (e.g. F, t, r) with confidence intervals, effect sizes, degrees of freedom and P value noted<br>*Give P values as exact values whenever suitable.*
☒	☐	For Bayesian analysis, information on the choice of priors and Markov chain Monte Carlo settings
☒	☐	For hierarchical and complex designs, identification of the appropriate level for tests and full reporting of outcomes
☒	☐	Estimates of effect sizes (e.g. Cohen's d, Pearson's r), indicating how they were calculated

Our web collection on statistics for biologists contains articles on many of the points above.

Software and code

Policy information about availability of computer code

Data collection	NA
Data analysis	XDS, CCP4 suite v7.0 (Phaser-MR, FFT, COOT), PHENIX v1.13, HOLE2 v2.2.005, MEGAX v10.1.8, Clustal Omega, PyMOL v2.3.2, Cuemol2 v2.2.3.443, Jaguar v7.9, NAMD v2.13, CHARMM v35b2, CHARMM-GUI v3.1

For manuscripts utilizing custom algorithms or software that are central to the research but not yet described in published literature, software must be made available to editors and reviewers. We strongly encourage code deposition in a community repository (e.g. GitHub). See the Nature Research guidelines for submitting code & software for further information.

Data

Policy information about availability of data

All manuscripts must include a data availability statement. This statement should provide the following information, where applicable:
- Accession codes, unique identifiers, or web links for publicly available datasets
- A list of figures that have associated raw data
- A description of any restrictions on data availability

The coordinates and structure factors for Lsi1cryst have been deposited in the Protein Data Bank (PDB) with accession number 7CJS. The source data for Figs. 2, 5, and 7, and Supplementary Figs. 3, 6, 7, 8, 10, 11, 12, and 16 have been provided as the Source Data file. Any other data associated with this manuscript are available from the authors at a reasonable request.

Field-specific reporting

Please select the one below that is the best fit for your research. If you are not sure, read the appropriate sections before making your selection.

☒ Life sciences ☐ Behavioural & social sciences ☐ Ecological, evolutionary & environmental sciences

For a reference copy of the document with all sections, see nature.com/documents/nr-reporting-summary-flat.pdf

Life sciences study design

All studies must disclose on these points even when the disclosure is negative.

Sample size	No statistical methods were used to predetermine sample sizes. The sample size was chosen so that intensities were measured by X-ray crystallography.
Data exclusions	No data was excluded unless unless rejected by the standard analysis software; for details of the data analysis statistics, see Methods and Table 1.
Replication	The structure was solved from several numbers of single crystals, and we confirmed the consistency. For the functional assays, we repeated several times and confirmed the consistency.
Randomization	Randomization is not applicable. The biological experiments in this study were carried out on purified protein or membrane samples that were validated in multiple ways.
Blinding	Due to the nature of the experimental setup, blinding was not applicable.

Reporting for specific materials, systems and methods

We require information from authors about some types of materials, experimental systems and methods used in many studies. Here, indicate whether each material, system or method listed is relevant to your study. If you are not sure if a list item applies to your research, read the appropriate section before selecting a response.

Materials & experimental systems

n/a	Involved in the study	
☐	☒	Antibodies
☐	☒	Eukaryotic cell lines
☒	☐	Palaeontology and archaeology
☐	☒	Animals and other organisms
☒	☐	Human research participants
☒	☐	Clinical data
☒	☐	Dual use research of concern

Methods

n/a	Involved in the study	
☒	☐	ChIP-seq
☒	☐	Flow cytometry
☒	☐	MRI-based neuroimaging

Antibodies

Antibodies used	Anti-green fluorescent protein tag rabbit polyclonal antibody-HRP-DirecT (1:2,000, MBL 598-7), anti-DYKDDDDK tag monoclonal antibody (1:1,000, Invitrogen MA1-91878), anti-Mouse IgG HRP Conjugate (1:20,000, Promega W4021), Monoclonal ANTI-FLAG® M2-Peroxidase (HRP) antibody produced in mouse (1:1,000, Sigma-Aldrich A8529).
Validation	All primary antibodies have been used to western blot either membrane fraction from Xenopus oocytes or lysates from Sf9 cells. The validation of the antibodies can be found on the manufacturer's website.

Eukaryotic cell lines

Policy information about cell lines

Cell line source(s)	The Sf9 cell line used in this study was obtained from thermo fisher science (catalog # 12659017).
Authentication	None of the cell line used was authenticated.
Mycoplasma contamination	The cell line was not tested for mycoplasma contamination. Sf9 cells were used solely for the purpose of producing recombinant proteins.
Commonly misidentified lines (See ICLAC register)	NA

Animals and other organisms

Policy information about studies involving animals; ARRIVE guidelines recommended for reporting animal research

Laboratory animals	No live animals were used in the study. Oocytes are obtained from frog, Xenopus laevis adult females.
Wild animals	This study does not involved wild animals.
Field-collected samples	Samples were not collected from the field.
Ethics oversight	The frog is commercially available and no ethical approval is required.

Note that full information on the approval of the study protocol must also be provided in the manuscript.
